# Supplementary material for: Comparison of Microalgae Nannochloropsis oceanica and Chlorococcum amblystomatis Lipid Extracts Effects on UVA-Induced Changes in Human Skin Fibroblasts Proteome
Source: Mar Drugs. 2024 Nov 10;22(11):509. doi: 10.3390/md22110509 (PMC11595653; doi:10.3390/md22110509)
Supplement: Supplementary file 1 [file marinedrugs-22-00509-s001.zip › Supplementary-6.11.2024.pdf]

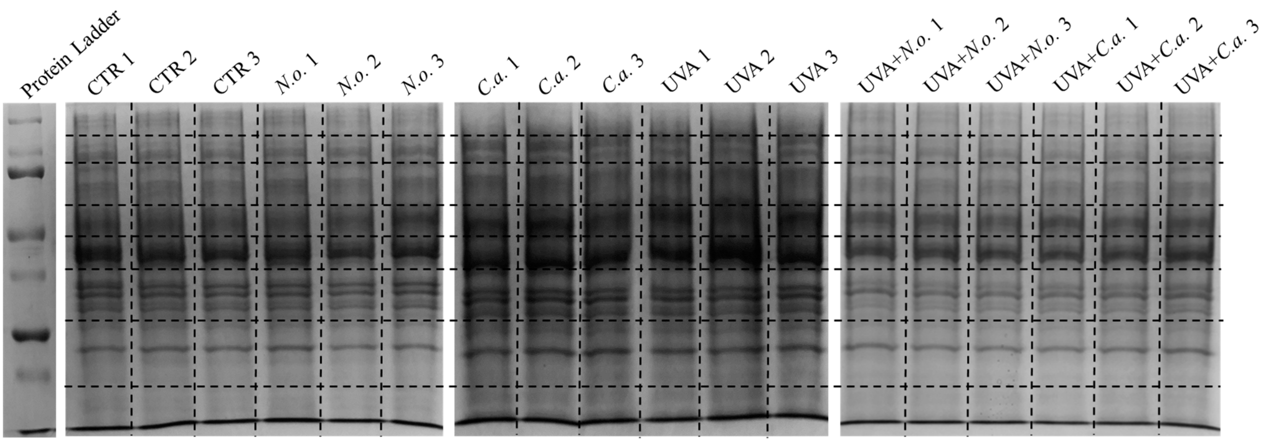

Supplementary Figure S1: SDS-PAGE gels stained by Coomassie brilliant blue. The samples with total proteins extracts from skin fibroblast loaded out in order are as follows: Control groups (**CTR** 1/2/3), the cells treated with lipid extract obtained from *N. oceanica* (***N.o.*** **1/2/3**), the cells treated with lipid extract obtained from *C. amblystomatis* (***C.a.*** **1/2/3**), the cells exposed to UVA radiation (**UVA 1/2/3**), the cells exposed to UVA radiation and treated with lipid extract obtained from *N. oceanica* (**UVA+*N.o.* 1/2/3**), and the cells exposed to UVA radiation and treated with lipid extract obtained from *C. amblystomatis* (**UVA+*C.a.* 1/2/3**). The way of gel cutting-slicing into eight sections for following in-gel trypsinization step is presented by dashed line.

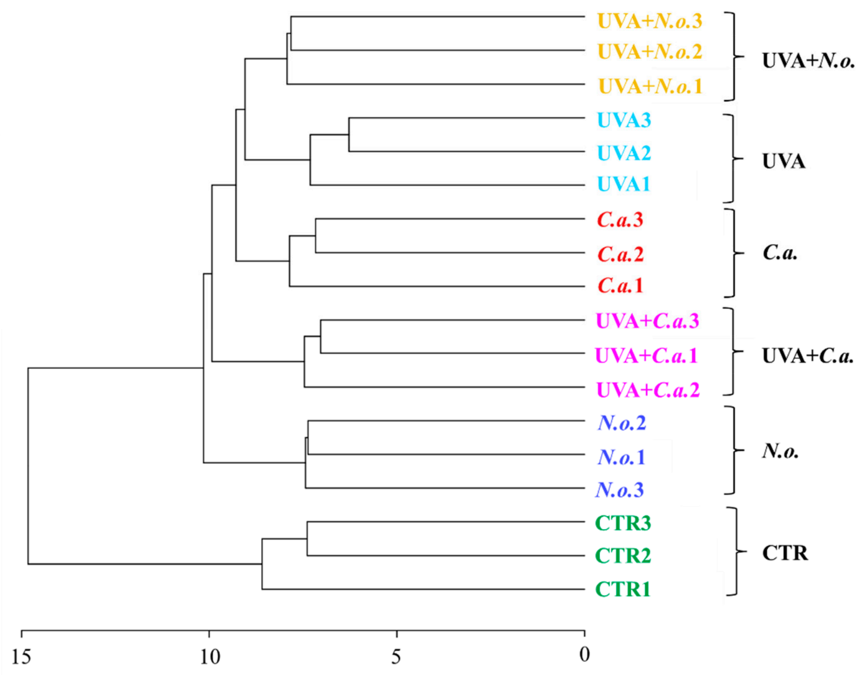

Supplementary Figure S2. The dendrogram created using all identified 494 proteins in the fibroblast cell groups (**CTR**, control cells cultured in the standard growing medium; ***N.o.***, cells cultured in the standard growing medium containing also lipid extract from *N. oceanica* (2µg/ml) for 24h; ***C.a.***, cells cultured in the standard growing medium containing lipid extracts from *C. amblystomatis* (2µg/ml) for 24h; **UVA**, cells exposed to UVA (365 nm) at a dose of 13 J/cm^3 and then incubated in the standard growing medium for 24h; **UVA+*N.o.***, cells exposed to UVA (365 nm) at a dose of 13 J/cm^3 and then incubated in the standard growing medium containing lipid extracts from *N. oceanica* (2µg/ml) for 24h; **UVA+*C.a.***, cells exposed to UVA (365 nm) at a dose of 13 J/cm^3 and then incubated in the standard growing medium containing lipid extracts from *C. amblystomatis* (2µg/ml) for 24h).

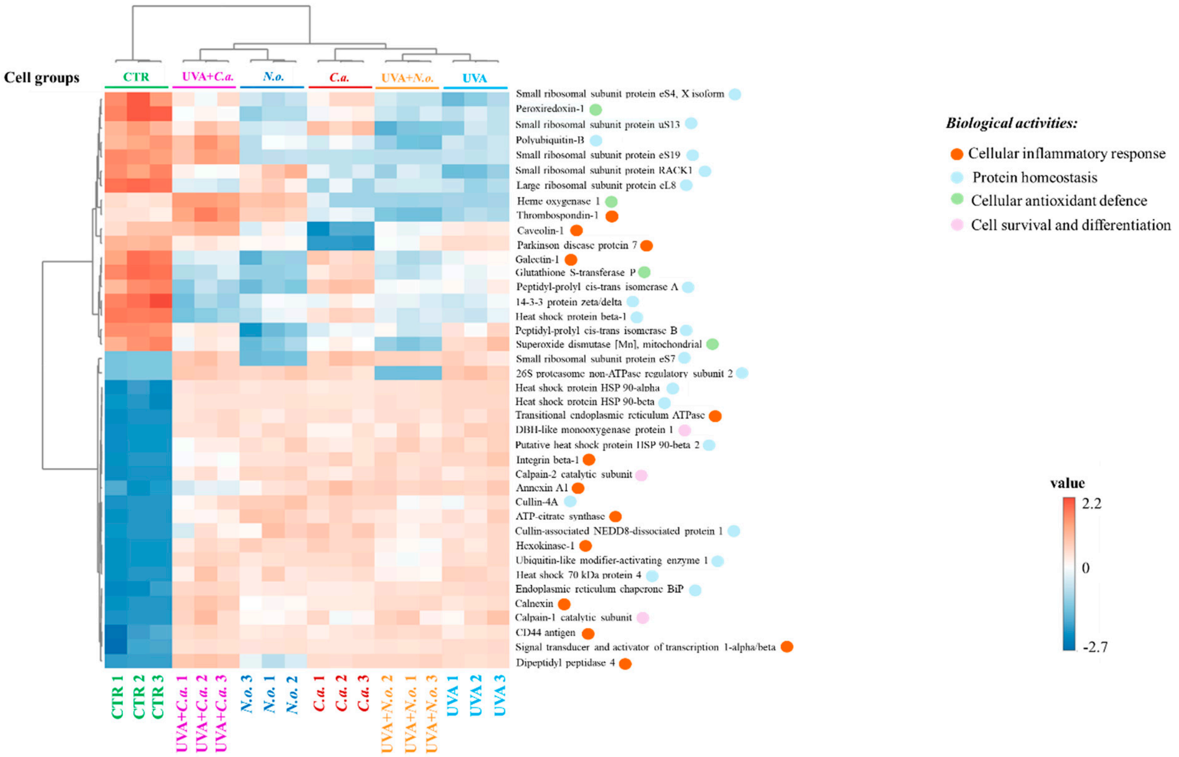

Supplementary Figure S3. Heatmap and clustering analysis of proteins of interest (i.e., those with significantly altered expression between fibroblast groups) involved in the cellular response to UVA-induced redox imbalance, with a focus on those regulating this response of cellular inflammatory response, protein homeostasis, cellular antioxidant defense or cell survival and differentiation. (**CTR**, control cells cultured in the standard growing medium; ***N.o.***, cells cultured in the standard growing medium containing also lipid extract from *N. oceanica* (2µg/ml) for 24h; ***C.a.***, cells cultured in the standard growing medium containing lipid extracts from *C. amblystomatis* (2µg/ml) for 24h; **UVA**, cells exposed to UVA (365 nm) at a dose of 13 J/cm^3 and then incubated in the standard growing medium for 24h; **UVA+*N.o.***, cells exposed to UVA (365 nm) at a dose of 13 J/cm^3 and then incubated in the standard growing medium containing lipid extracts from *N. oceanica* (2µg/ml) for 24h; **UVA+*C.a.***, cells exposed to UVA (365 nm) at a dose of 13 J/cm^3 and then incubated in the standard growing medium containing lipid extracts from *C. amblystomatis* (2µg/ml) for 24h)

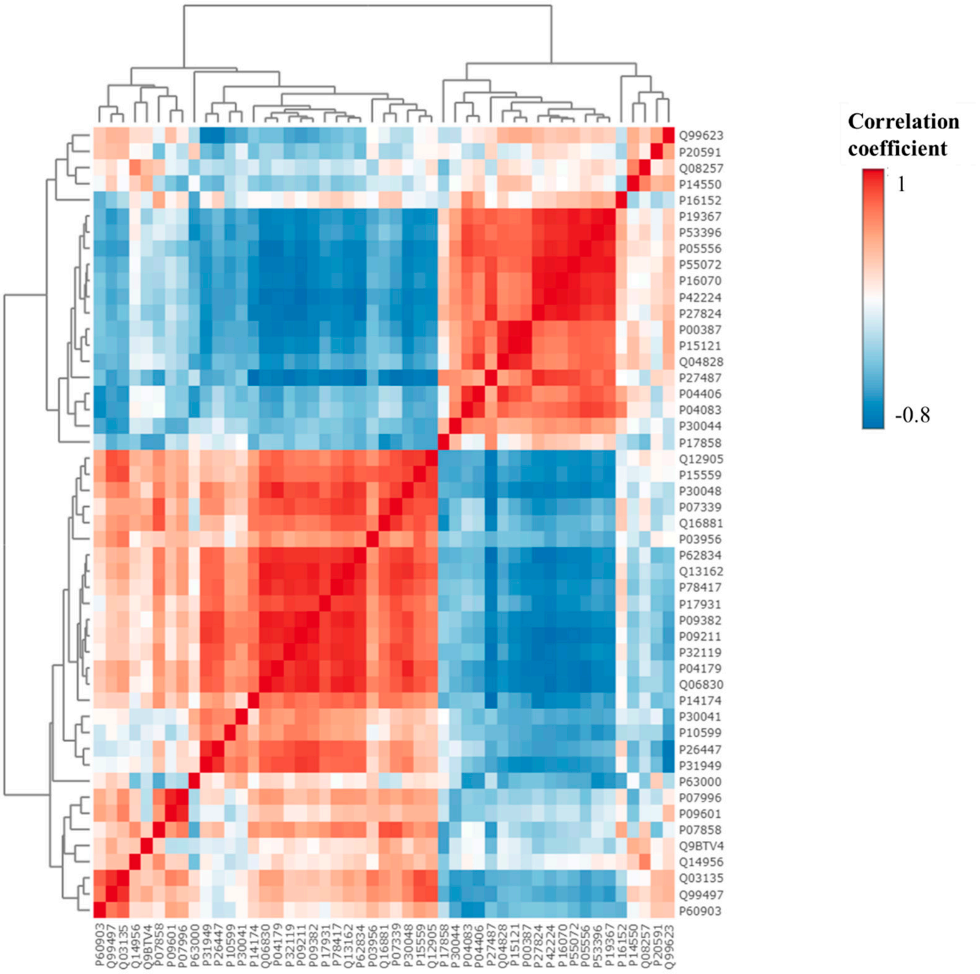

Supplementary Figure S4. Heatmap of Pearson correlation matrix of identified proteins showing significantly changed expression between cell groups (**CTR**, ***N.o.***, ***C.a.***, **UVA**, **UVA+*N.o.***, **UVA+*C.a.***, as explained in **Table 1**) and participating in regulation of intracellular redox balance or inflammatory cellular response.

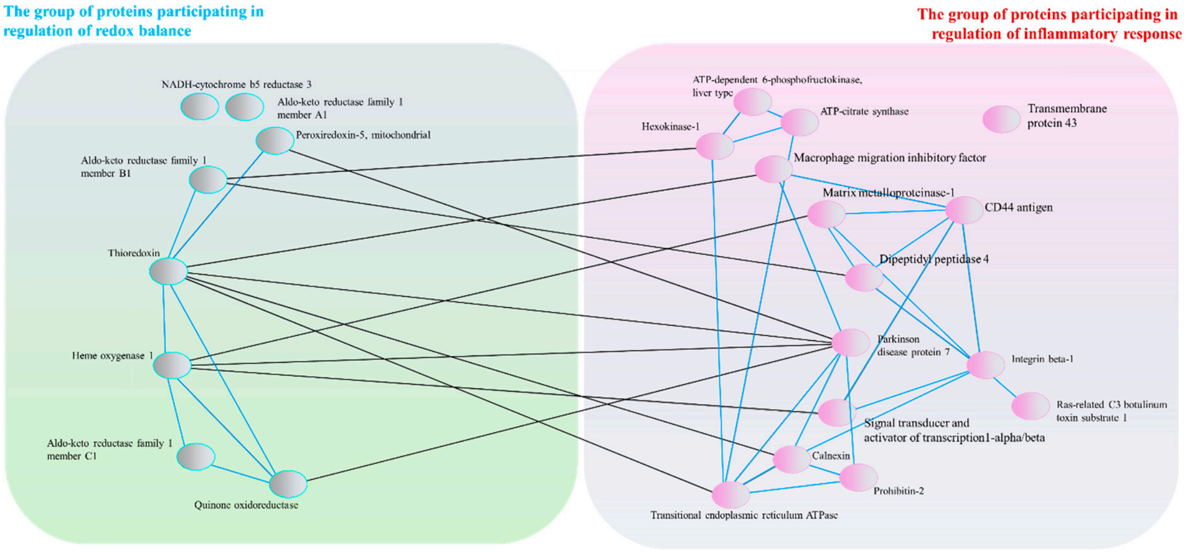

Supplementary figure S5. Protein – protein interactions among proteins with significantly altered expression between fibroblast groups (**CTR**, ***N.o.***, ***C.a.***, **UVA**, **UVA+*N.o.***, **UVA+*C.a.***, as explained in **Table 1**). Black line used for highlighting interaction between the proteins participating in regulation of redox balance and the proteins participating in regulation of inflammatory response. While blue lines used for indicating the interaction belonging to the same group.

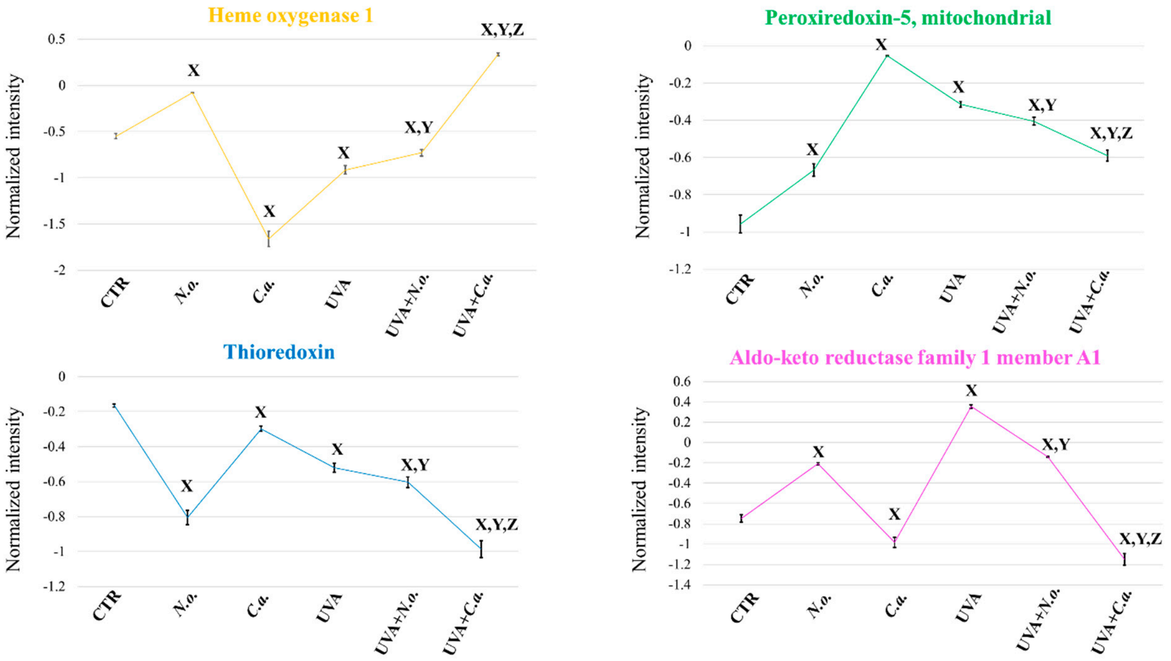

Supplementary figure S6. Profile plot generated using average intensities (log-transformed and normalized by median) of proteins with cytoprotective activity against oxidative stress which their levels were significantly changed between cell groups (**CTR**, control cells cultured in the standard growing medium; ***N.o.***, cells cultured in the standard growing medium containing also lipid extract from *N. oceanica* (2µg/ml) for 24h; ***C.a.***, cells cultured in the standard growing medium containing lipid extracts from *C. amblystomatis* (2µg/ml) for 24h; **UVA**, cells exposed to UVA (365 nm) at a dose of 13 J/cm^3 and then incubated in the standard growing medium for 24h; **UVA+*N.o.***, cells exposed to UVA (365 nm) at a dose of 13 J/cm^3 and then incubated in the standard growing medium containing lipid extracts from *N. oceanica* (2µg/ml) for 24h; **UVA+*C.a.***, cells exposed to UVA (365 nm) at a dose of 13 J/cm^3 and then incubated in the standard growing medium containing lipid extracts from *C. amblystomatis* (2µg/ml) for 24h). Mean values ± SD of three independent samples and statistically significant differences for $p \leq 0.05$ are presented: (**X**) is used for differences vs. CTR; (**Y**) is used for differences between the group UVA and UVA+*N.o.* or UVA+*C.a.*; (**Z**) is used for differences between UVA+*N.o.* and UVA+*C.a.*

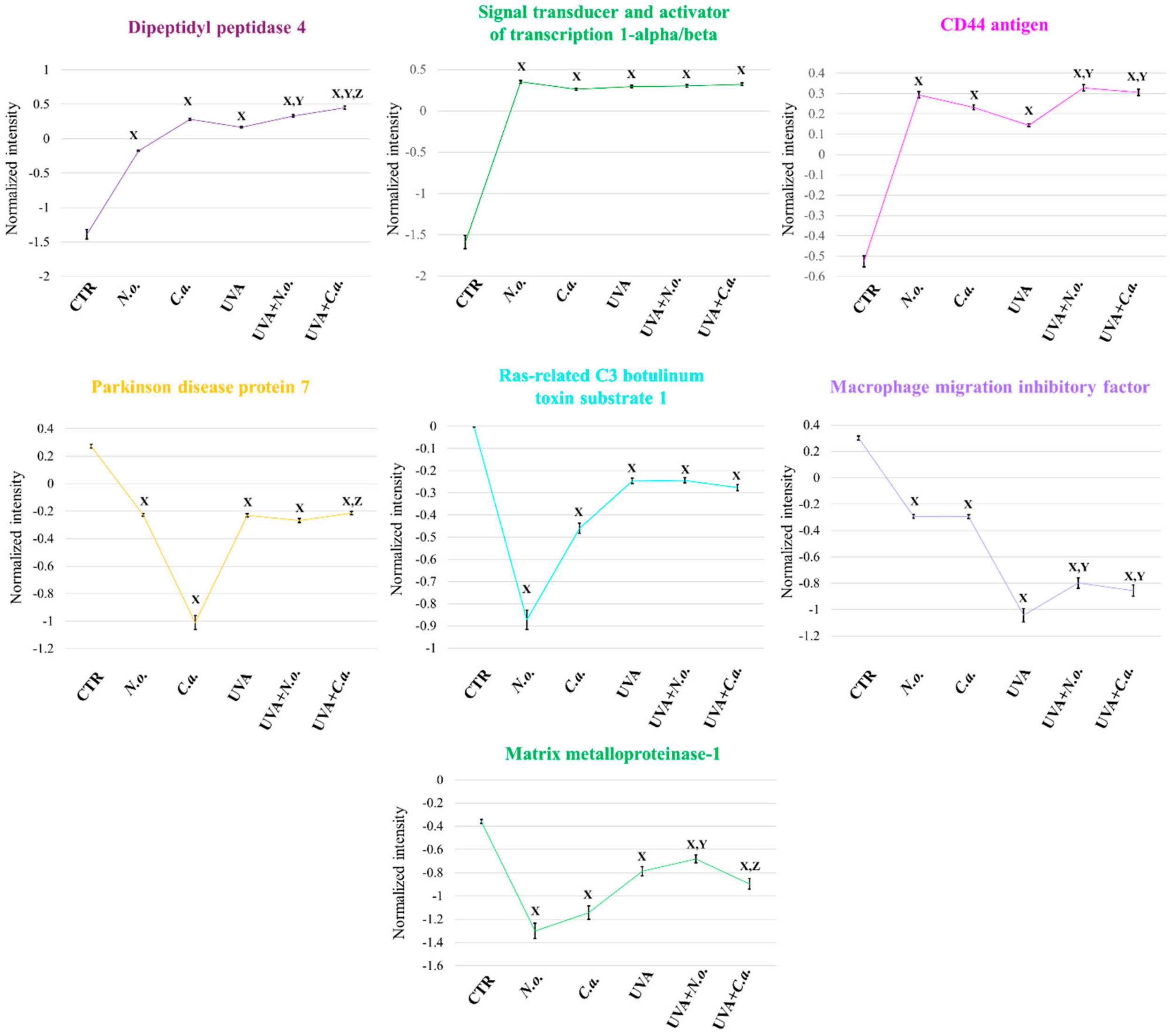

Supplementary figure S7. Profile plot generated using average intensities (log-transformed and normalized by median) of proteins participating in the regulation of cellular inflammatory response which their levels were significantly changed between cell groups (**CTR**, control cells cultured in the standard growing medium; ***N.o.***, cells cultured in the standard growing medium containing also lipid extract from *N. oceanica* (2µg/ml) for 24h; ***C.a.***, cells cultured in the standard growing medium containing lipid extracts from *C. amblystomatis* (2µg/ml) for 24h; **UVA**, cells exposed to UVA (365 nm) at a dose of 13 J/cm^3 and then incubated in the standard growing medium for 24h; **UVA+*N.o.***, cells exposed to UVA (365 nm) at a dose of 13 J/cm^3 and then incubated in the standard growing medium containing lipid extracts from *N. oceanica* (2µg/ml) for 24h; **UVA+*C.a.***, cells exposed to UVA (365 nm) at a dose of 13 J/cm^3 and then incubated in the standard growing medium containing lipid extracts from *C. amblystomatis* (2µg/ml) for 24h). Mean values ± SD of three independent samples and statistically significant differences for $p \leq 0.05$ are presented: (**X**) is used for differences vs. CTR; (**Y**) is used for differences between the group UVA and UVA+*N.o.* or UVA+*C.a.*; (**Z**) is used for differences between UVA+*N.o.* and UVA+*C.a.*
